# MiR-497 suppresses angiogenesis and metastasis of hepatocellular carcinoma by inhibiting VEGFA and AEG-1

**DOI:** 10.18632/oncotarget.5012

**Published:** 2015-08-21

**Authors:** Jing-Jun Yan, Yu-Nan Zhang, Jia-Zhi Liao, Kun-peng Ke, Ying Chang, Pei-Yuan Li, Min Wang, Ju-Sheng Lin, Xing-Xing He

**Affiliations:** ^1^ Institute of Liver Diseases, Tongji Hospital, Tongji Medical College, Huazhong University of Science and Technology, Wuhan 430030, China; ^2^ Department of Emergency Medicine, Tongji Hospital, Tongji Medical College, Huazhong University of Science and Technology, Wuhan 430030, China; ^3^ Department of Cardiac Surgery, Wuhan Asia Heart Hospital, Wuhan 430022, China

**Keywords:** hepatocellular carcinoma, microRNA, tumor biology, cancer

## Abstract

Hepatocellular carcinoma (HCC) is a worldwide malignance and displays marked vascular abnormalities and active metastasis. MicroRNAs (miRNAs) have been shown to play important roles in regulating tumor properties in cancer, however, whether miR-497 contributes to HCC angiogenesis or metastasis remains unclear. In this study, we found that miR-497 was significantly down-regulated in HCC tissue samples and cell lines. Gain-of-function and loss-of-function studies revealed that miR-497 could repress both the pro-angiogenic and metastatic ability of HCC cells. Subsequent investigations disclosed that miR-497 directly inhibited the 3′-untranslated regions (UTRs) of vascular endothelial growth factor A (VEGFA) and astrocyte elevated gene-1 (AEG-1). Furthermore, overexpression of these targets antagonized the function of miR-497. Based on nude mouse models, we demonstrated that overexpression of miR-497 significantly repressed microvessel densities in xenograft tumors and reduced pulmonary metastasis. In conclusion, our findings indicate that miR-497 downregulation contributes to angiogenesis and metastasis in HCC.

## INTRODUCTION

Hepatocellular carcinoma (HCC) is the second leading cause of cancer-related deaths worldwide, with approximately 750,000 new cases of liver cancer reported per year [1]. The heavy burden of HCC has generated extensive studies of molecular mechanisms underlying this disease. Abnormal angiogenesis and frequent metastasis are two important hallmarks of HCC [2]. The capability of angiogenesis addresses the needs of tumor for nutrients and oxygen, while the ability of metastasis enables cancer cells to colonize new terrain to obtain more nutrients and spaces [2]. Therefore, novel anticancer strategies focused on molecules that possess antiangiogenic or antimetastatic activities may provide promising breakthroughs for HCC treatment.

MicroRNAs (miRNAs) are a class of endogenous and small noncoding regulatory RNAs, which mainly recognize complementary sequences in the 3′-untranslated regions (UTRs) of their target genes and leading to mRNA degradation or translation inhibition, and recent studies reported that they can also bind to the 5′-UTR or the open reading frame (ORF) and upregulate translation upon growth arrest conditions [3–6]. Accumulating evidence suggested that miRNAs could act as oncogenes or tumor suppressors by regulating target genes involved in cell cycle, proliferation, apoptosis, metastasis and angiogenesis [7]. Recently, the expression level of miR-497 was reported to be reduced in multiple types of cancers including renal cancer [8], ovarian cancer [9], pancreatic cancer [10] as well as HCC [11]. In HCC, previous studies have shown that miR-497 blocked cell cycle at G1 phase by suppressing the expression of CCNE1, CDC25A, CCND3, CDK4, BTRC, and Checkpoint kinase 1 [11, 12]. However, whether the dysregulation of miR-497 contributes to HCC angiogenesis or metastasis remains unclear.

Previously, we have also identified a group of differentially expressed miRNAs between cancerous hepatocytes and normal primary human hepatocytes through human microRNA arrays, and found that miR-497 was one of the significantly down-regulated miRNAs [13]. Interestingly, predicated target genes of miR-497 including vascular endothelial growth factor A (VEGFA) and astrocyte elevated gene-1 (AEG-1), which play pivotal roles in angiogenesis and metastasis in HCC respectively [14–16]. During the further characterization of miR-497 in HCC, we found that miR-497 functioned as an angiogenesis and metastasis suppressor. Restoration of miR-497 in HCC cells significantly suppressed tumor angiogenesis and metastasis *in vitro* and *in vivo*. Subsequent experiments confirmed that miR-497 exerted its anti-angiogenic and anti-metastatic effects by directly inhibiting VEGFA and AEG-1.

## RESULTS

### MiR-497 is down-regulated in HCC cells and tissues

**Figure 1:**
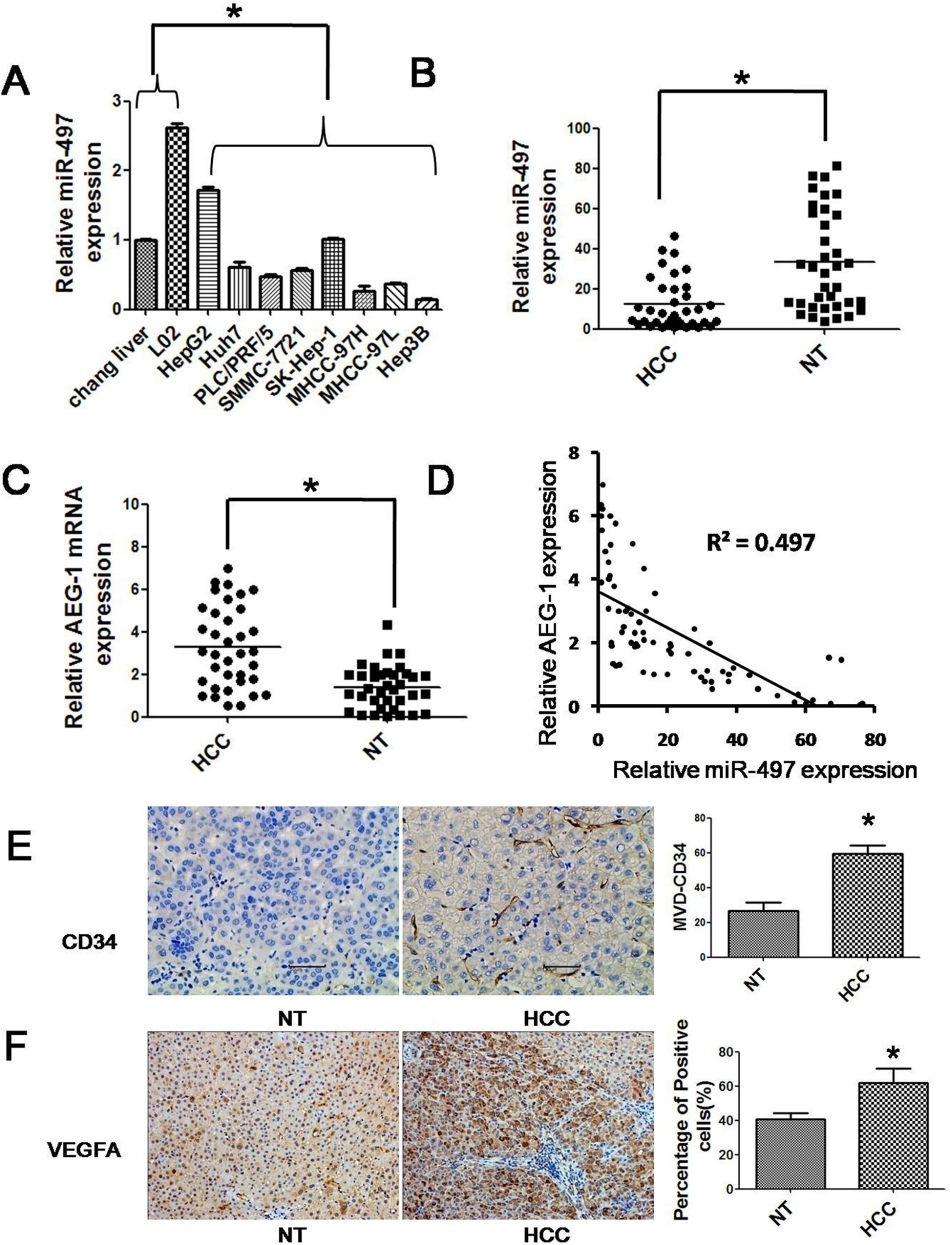
Reduced expression of miR-497 in liver cancer cell lines and tissues **A.** The expressions of miR-497 in eight human liver cancer cell lines (HepG2, Huh7, PLC/PRF/5, SMMC-7721, SK-HEP-1, MHCC97-H, MHCC97-L and Hep3B) and two human normal hepatic cell lines (Chang liver and L02) were measured by SYBR Green qRT-PCR. **B.** miR-497 (measured by SYBR Green qRT-PCR) was down-regulated in HCC tissues compared with the matched adjacent non-tumorous liver tissues. The central horizontal line represents the mean value. **C.** AEG-1 (measured by SYBR Green qRT-PCR) was up-regulated in HCC tissues compared with the matched adjacent non-tumorous liver tissues. The central horizontal line represents the mean value. **D.** AEG-1 was inversely correlated with miR-497 expression in the human HCC tissues. **E.** and **F.** The expression of CD34 and VEGFA was measured by immunohistochemical staining in the human HCC tissues and matched adjacent non-tumorous liver tissues. HCC: hepatocellular carcinoma tissues; NT: matched adjacent non-tumorous liver tissues; MVD: microvessel density. *P < 0.05.

We previously identified a specific miRNA expression profiling in liver cancer and the microarray data were deposited in NCBI's Gene Expression Omnibus (GEO) public database (http://www.ncbi.nlm.nih.gov/geo/, GEO accession number, GSE20077) [13]. In this study, we examined the expression levels of miR-497 in eight hepatoma cell lines (HepG2, Huh7, PLC/PRF/5, SMMC-7721, SK-HEP-1, MHCC97-H, MHCC97-L and Hep3B) and two normal hepatic cell lines (Chang liver and L02) by SYBR Green qRT-PCR to assess the microarray data. In accordance with the microarray results, the expression of miR-497 was lower in HCC cells than in normal hepatic cell lines (Figure 1A). Among the eight HCC cell lines, the expression of miR-497 in Huh7, PLC/PRF/5, SMMC-7721, MHCC-97H, MHCC-97L and Hep3B was much lower than its expression in HepG2 and SK-HEP-1 (Figure 1A). Next, we examined miR-497 expression levels in 36 pairs of HCC tissues and the corresponding adjacent noncancerous tissues. Consistently, miR-497 was also significantly suppressed in HCC tissues (Figure 1B). Interestingly, the abundance of miR-497 was inversely correlated with that of AEG-1 (Figures 1C and 1D), a potential target of miR-497. Microvessel density (MVD) is a commonly used parameter to assess the number of neovessels formed in the tumor tissues. Here, the level of MVD was quantified by immunohistochemical staining using endothelial cell markers CD34. As shown in Figure 1E, significant higher level of CD34 was detected in the tumor tissue than the adjacent nontumorous tissue. Besides, the expression of VEGFA, another potential target gene of miR-497, was significantly higher in HCC tissues than that in matched adjacent non-tumor tissues (Figure 1F).

### miR-497 suppresses pro-angiogenic and metastasis activity of HCC cells

**Figure 2:**
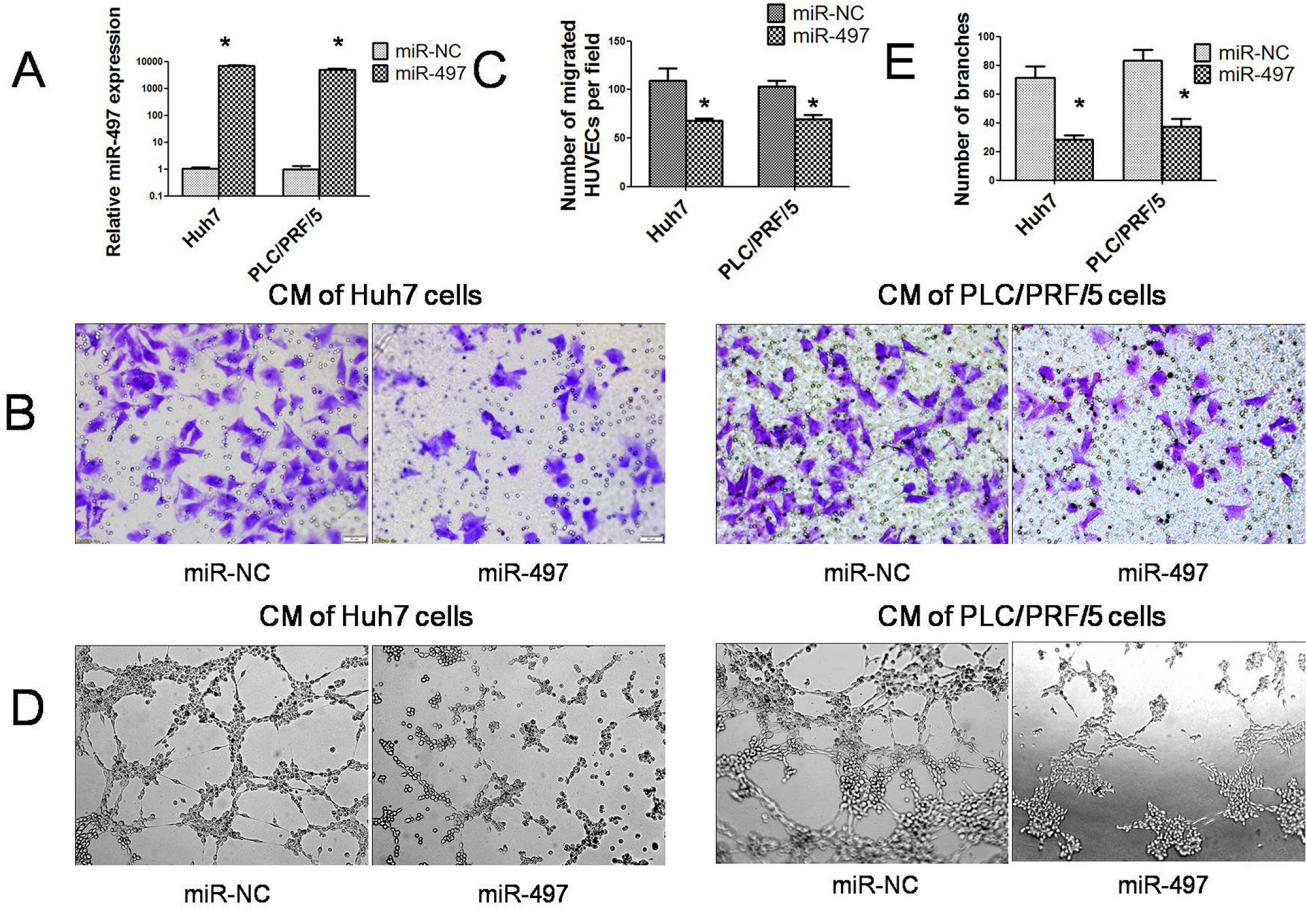
MiR-497 exerts antiangiogenic activity *in vitro* **A.** Relative expression of miR-497 was detected by SYBR Green qRT–PCR in two hepatoma cell lines (Huh7 and PLC/PRF/5) transfected with miR-NC or miR-497 mimic. The average miRNA expression in miR-NC group was designated as 1. **B.** Human umbilical vein endothelial cell (HUVEC) migration was evaluated using a 24-transwell chamber with 8 μm pore insert. The migration ability of HUVEC cells were significantly inhibited by miR-497 overexpression in both Huh7 and PLC/PRF/5 cells. (Olympus DP70, magnification ×200). **C.** Quantification of the migration capability of HUVEC cells. **D.** Restoration of miR-497 suppressed the HCC cell–promoted HUVEC tube formation. (Olympus DP70, magnification ×100) **E.** Quantification of the branches formed by HUVEC cells. CM: conditional medium; *P < 0.05.

To explore the effect of miR-497 on HCC angiogenesis, *in vitro* endothelial recruitment and capillary tube formation assays were performed with two HCC cell lines, Huh7 and PLC/PRF/5. As shown in Figure 2A, the significant increasing of miR-497 expression could be verified by SYBR Green qRT-PCR in hepatoma cell lines transfected with 50nM miR-497 mimics. The endothelial recruitment assay, performed in 24-transwell chamber with 8 μm pore insert, revealed that the restoration of miR-497 expression significantly suppressed the ability of HCC cells to promote human umbilical vein endothelial cell (HUVEC) migration (Figure 2B and 2C). In addition, by using capillary tube formation assays, we observed that the morphological differentiation of HUVEC cells was affected by miR-497 overexpression in HCC cells (Figure 2D and 2E). HUVEC cells formed incomplete and fluffy tubular structures in the presence of CM obtained from miR-497 transfected HCC cells. In contrast, the treatment with CM obtained from negative control led to the formation of elongated and robust tubular structure. These results indicated that overexpression of miR-497 in HCC cells could inhibit pro-angiogenic activity of HCC cells *in vitro*.

**Figure 3:**
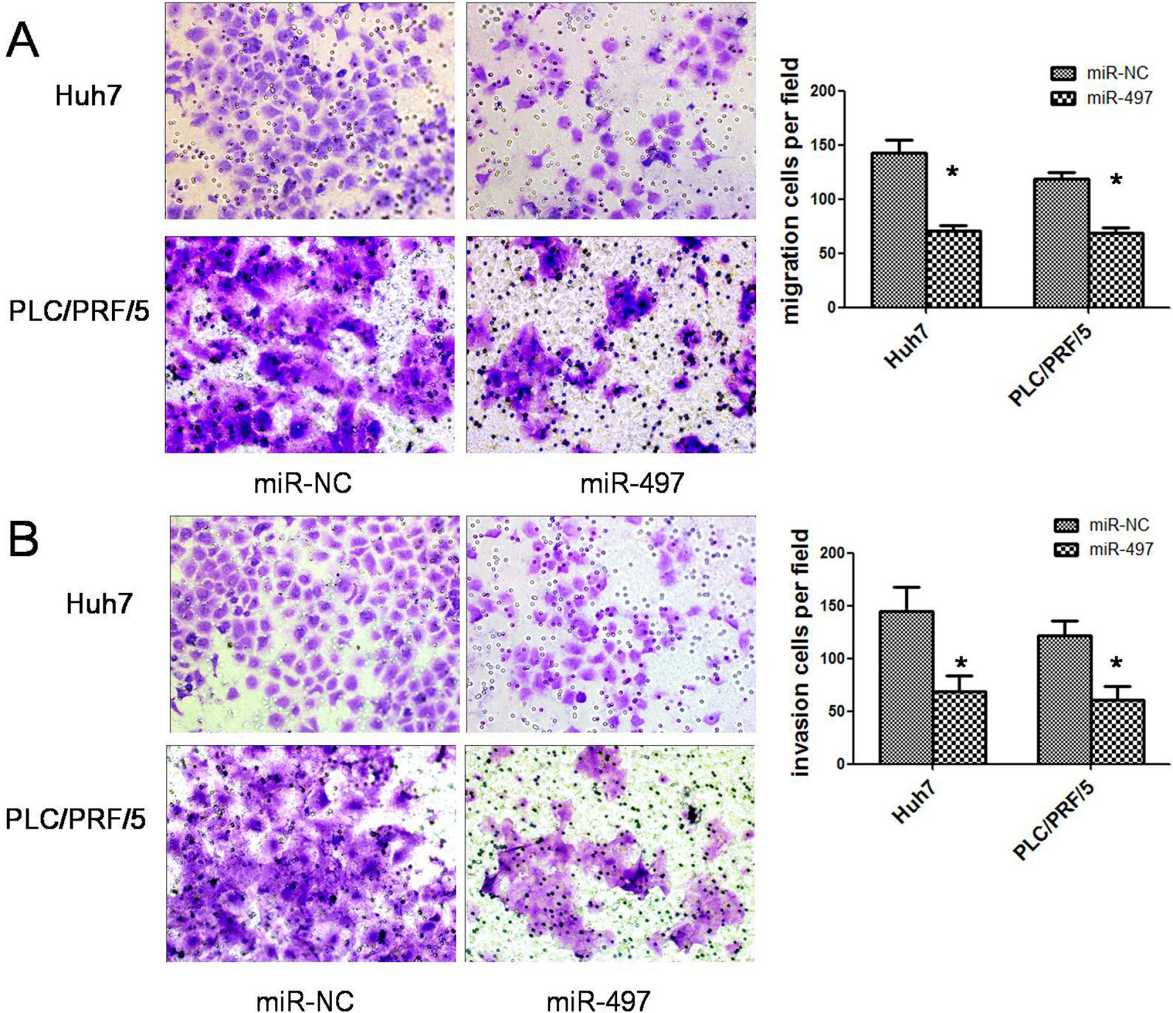
MiR-497 suppresses HCC migration and invasion *in vitro* **A.** Restoration of miR-497 inhibited HCC cell migration. Huh7 (upper) and PLC/PRF/5 (lower) cells that were transfected with miR-NC (left) or miR-497 (right) mimic were added to transwell chambers and incubated for 24 hours (Huh7) or 36 h (PLC/PRF/5), followed by staining with crystal violet. **B.** Restoration of miR-497 inhibited HCC cell invasion. Huh7 (upper) and PLC/PRF/5 (lower) cells that were transfected with miR-NC (left) or miR-497 (right) mimic were added to transwell chambers with Matrigel coatings and incubated for 24 hours (Huh7) or 36 h (PLC/PRF/5), followed by staining with crystal violet. Representative pictures and quantification of cells were shown. *P < 0.05.

Subsequently, to clarify the role of miR-497 in HCC metastasis, we analyzed the effects of miR-497 on the migration and invasion ability of HCC cells. Using transwell assays, we observed that both the migratory (Figure 3A) and invasive (Figure 3B) activities of HCC cells were suppressed by miR-497 overexpression.

### miR-497 inhibits HCC angiogenesis by directly targeting VEGFA

**Figure 4:**
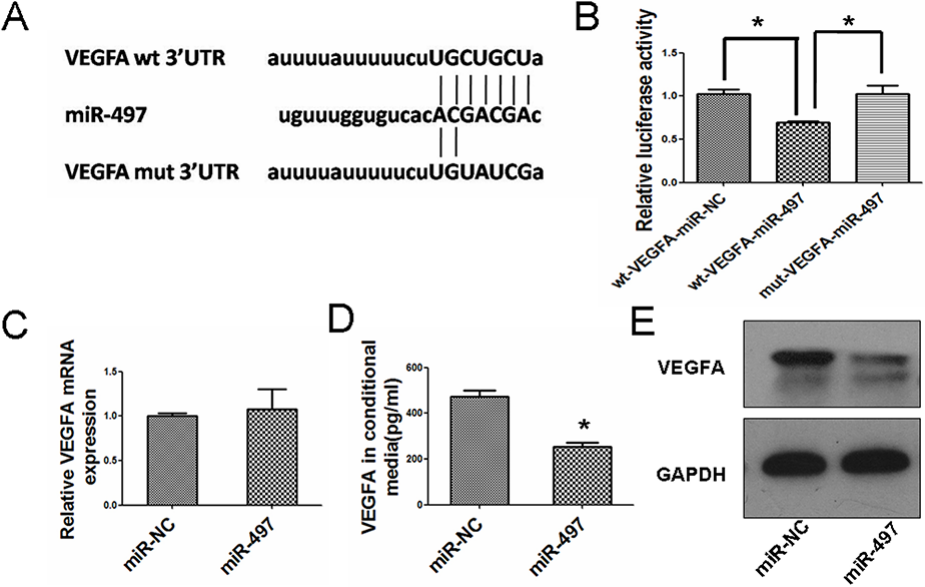
MiR-497 inhibits VEGFA expression by targeting its 3′UTR **A.** Putative binding sequence between the 3′UTR of VEGFA and miR-497. Mutations were generated in the complementary site that binds to the seed region of miR-497. **B.** pLUC-wt-VEGFA or pLUC-mut-VEGFA vectors were cotransfected with miR-497 mimic or miR-NC. Relative repression of luciferase expression was standardized to β-gal signal. Luciferase activity in pLUC-wt-VEGFA group displayed a significant decrease following ectopic expression of miR-497. **C.** mRNA relative expression measured by SYBR Green qRT-PCR at 48 h after transfection. The mRNA expression remained similar between the two groups. **D.** The amount of secreted VEGFA was decreased by restoration of miR-497. Conditional medium from Huh7 cells that were transfected with miR-NC or miR-497 mimic were analyzed by ELISA. **E.** VEGFA protein was downregulated in Huh7 cells transfected with miR-497 mimic. VEGFA protein was measured by immunoblotting at 48 h after transfection. GAPDH was used as an internal control. wt, wide type; mut, mutant type; *P < 0.05.

**Figure 5:**
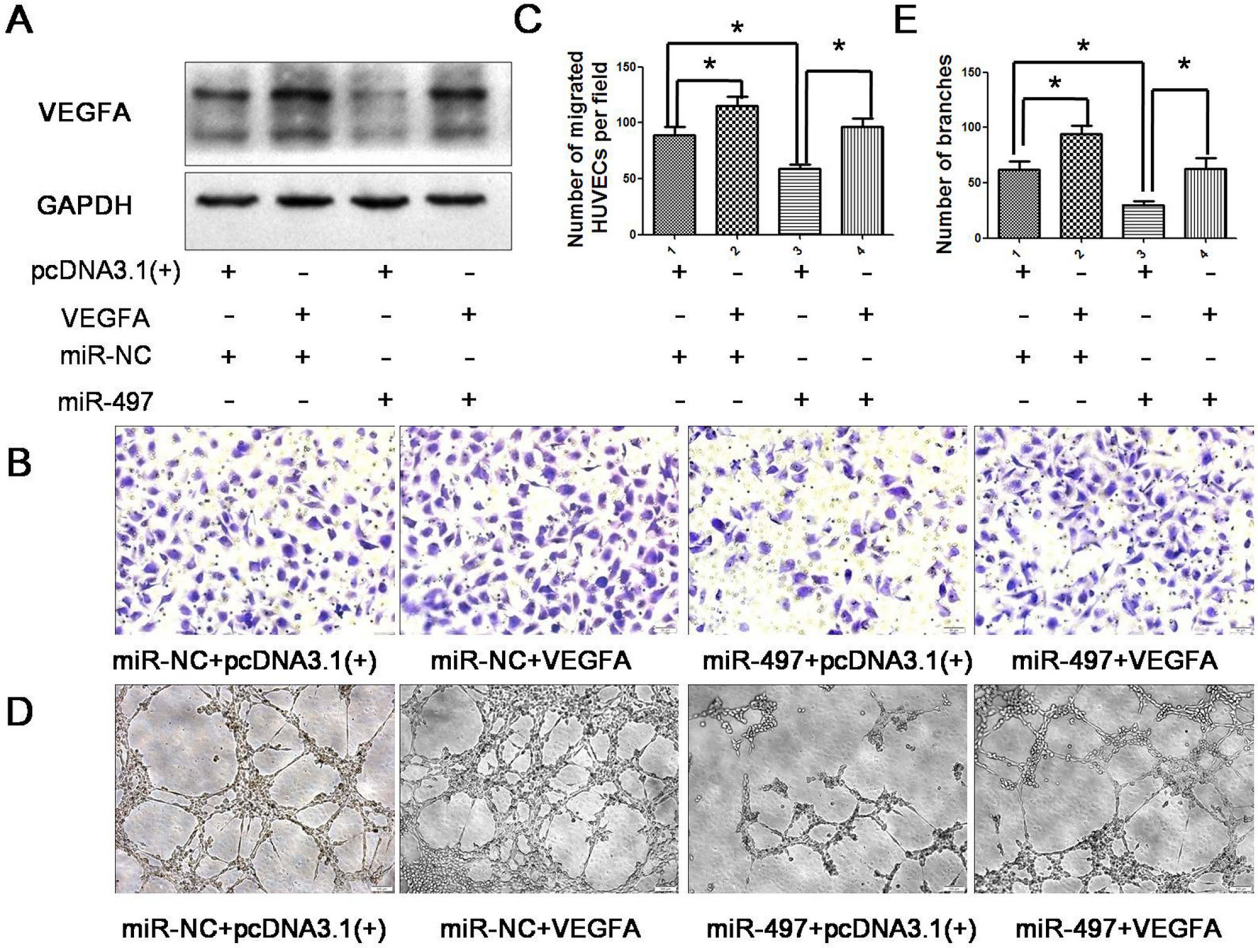
Over-expression of VEGFA attenuates the anti-angiogenic effect of miR-497 **A.** Forty-eight hours after co-transfected with the following RNA duplex/expression plasmid combinations: miR-NC/empty vector pcDNA3.1(+) (*panel* 1), miR-NC/VEGFA (*panel* 2), miR-497/ pcDNA3.1 (*panel* 3) or miR-497/VEGFA (*panel* 4), Huh7 cells were analyzed by immunoblotting. GAPDH was used as an internal control. **B.** Human umbilical vein endothelial cell (HUVEC) migration was evaluated using a 24-transwell chamber with 8 μm pore insert. The inhibition of migration ability of HUVEC cells by miR-497 overexpression was antagonized by introduction of VEGFA (Olympus DP70, magnification ×200). **C.** Quantification of the migration capability of HUVEC cells. **D.** Introduction of VEGFA antagonized the anti-tube formation effect of miR-497. HUVECs were cultured in conditional medium derived from Huh7 cells that were co-transfected RNA duplex/expression plasmid combinations. The capillary tube formation of HUVECs was assessed subsequently (Olympus DP70, magnification ×100). **E.** Quantification of the tube formation capability of HUVEC cells. The results were reproduced in three independent experiments, and representative images are shown. *P < 0.05.

To unravel the mechanism underlying miR-497 disrupted angiogenesis, we searched for positive regulators of angiogenesis using miRNA target prediction software (TargetScan and microRNA.org). VEGFA was found to be among the predicted high confidence targets (Figure 4A), and was chosen for further validation due to its well-known importance in tumor angiogenesis. Luciferase assay revealed that co-transfection of miR-497 significantly inhibited the activity of luciferase reporter with wild-type 3′UTR of VEGFA, whereas this effect was abrogated when the predicted 3′UTR binding site was mutated (Figure 4B). SYBR Green qRT-PCR result indicated that enhancing miR-497 expression had no obviously effects on the mRNA level of VEGFA (Figure 4C). Then, enzyme-linked immunosorbent assay test was performed to determine whether the secretion of VEGFA protein was altered after miR-497 transfection. As shown in Figure 4D, VEGFA protein levels in miR-497 transfected cell culture supernatant were significantly decreased compared with those transfected with miR-NC. Furthermore, western blot analysis also showed that the restoration expression of miR-497 in Huh7 cells reduced the expression of VEGFA at the protein level (Figure 4E). These data indicated that miR-497 may negatively regulate VEGFA expression at the translational level by directly targeting its 3′UTR. We further validated whether VEGFA mediates the angiogenic function of HCC. Suppression of VEGFA in HCC cells displayed a significantly reduced capacity to promote HUVEC migration and capillary tube formation, which phenocopied the effects of miR-497 overexpression (Supplementary Figure S1). Consistently, the overexpression of VEGFA in miR-497 transfectants obviously abrogated the inhibitory effects of miR-497 on HUVEC migration and capillary tube formation in Huh7 cells (Figure 5), while down-regulation of VEGFA in anti-miR-497 transfectants obviously abrogated the pro-angiogenic effects of anti-miR-497 on HepG2 cells (Supplementary Figure S2). These results suggested that miR-497 repressed tumor angiogenesis by inhibiting VEGFA in HCC cells.

### miR-497 represses HCC metastasis by negatively regulating AEG-1 expression

**Figure 6:**
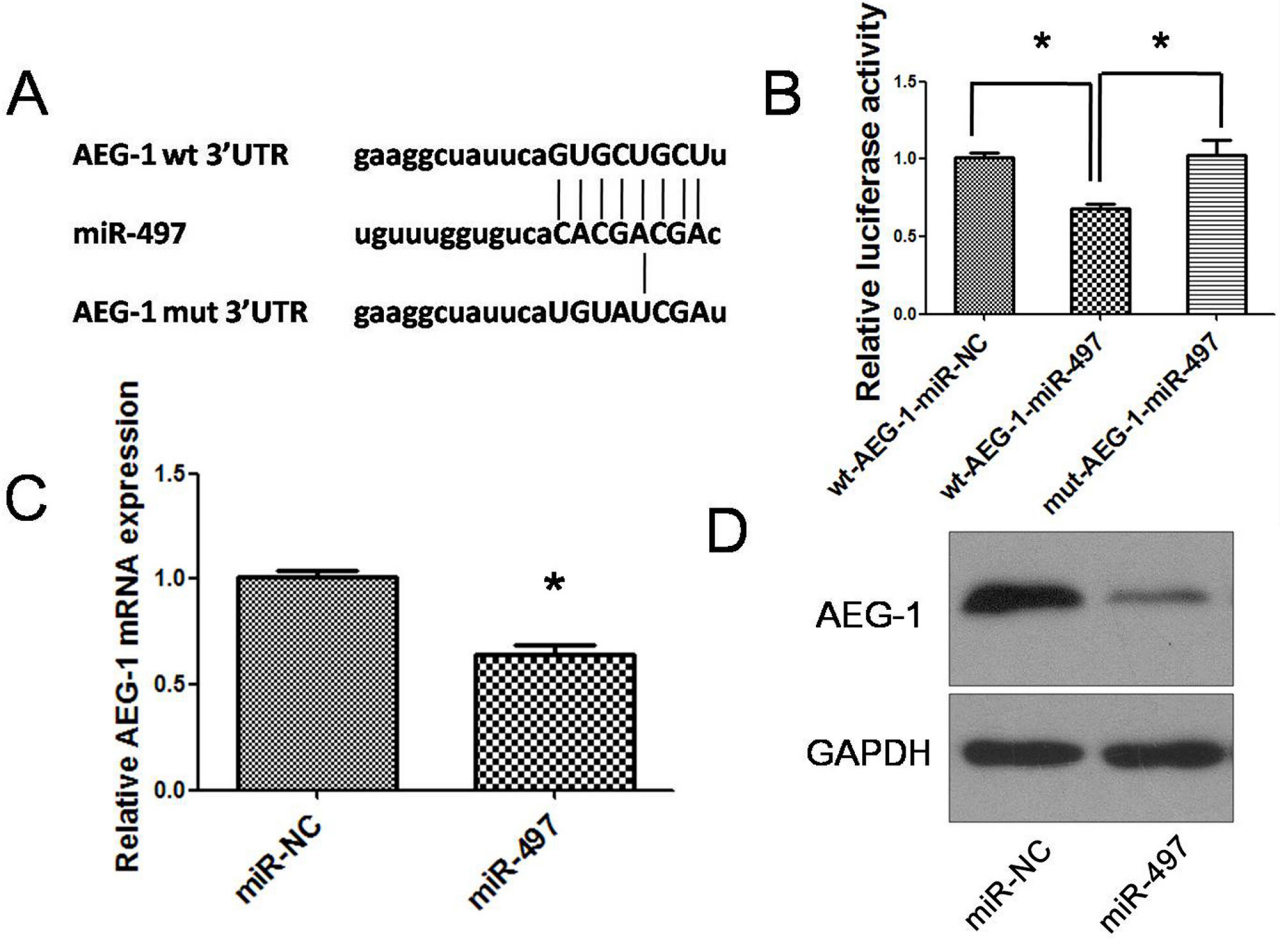
MiR-497 directly targets AEG-1 in hepatocarcinoma **A.** miR-497 and its putative binding sequences in the 3′UTR of AEG-1. Mutations were generated in the complementary sites that bind to the seed region of miR-497. **B.** pLUC-wt-AEG-1 or pLUC-mut-AEG-1 vector was cotransfected with miR-NC or miR-497 mimics. Relative repression of luciferase expression was standardized to β-gal signal. Luciferase activity in pLUC-wt-AEG-1 group displayed a significant decrease following ectopic expression of miR-497. **C.** AEG-1 mRNA was downregulated in Huh7 cells transfected with miR-497 mimic. **D.** Expression of miR-497 reduced the protein levels of cellular AEG-1. Huh7 cells that were transfected with miR-NC or miR-497 for 48 h were analyzed by western blot assay. GAPDH was used as an internal control. wt, wide type; mut, mutant type; *P < 0.05.

**Figure 7:**
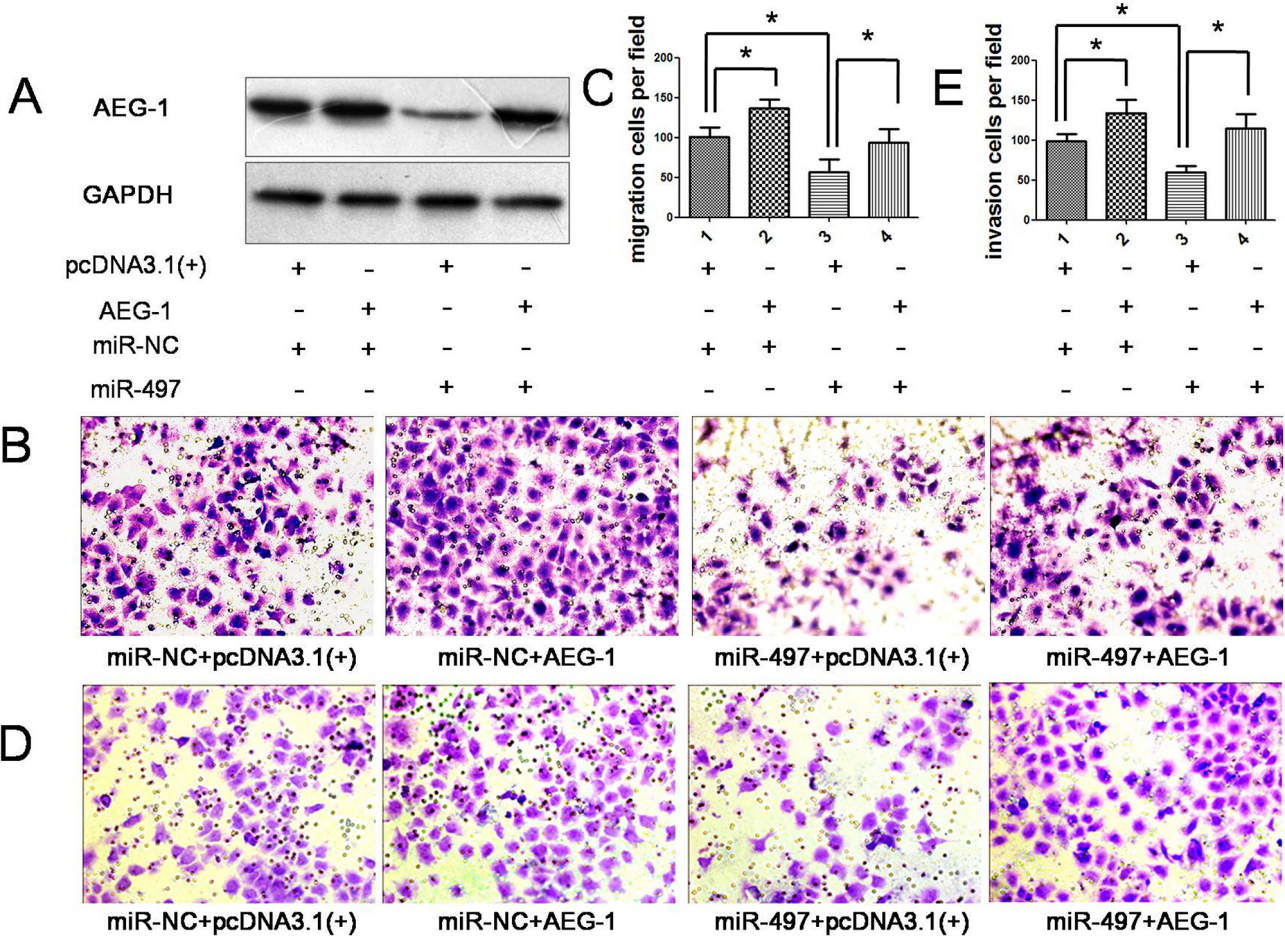
Overexpression of AEG-1 attenuated the anti-metastatic effect of miR-497 **A.** Forty-eight hours after co-transfected with the following RNA duplex/expression plasmid combinations: miR-NC/empty vector pcDNA3.1(+) (panel 1), miR-NC/ AEG-1 (panel 2), miR-497/ pcDNA3.1 (panel 3) or miR-497/ AEG-1 (panel 4), expression of AEG-1 protein in Huh7 cells were analyzed by immunoblotting. GAPDH was used as an internal control. **B.** and **C.** Introduction of AEG-1 antagonized the anti-migration effect of miR-497 (Olympus DP70, magnification ×200). **D.** and **E.** Overexpression of AEG-1 rescue the anti-invasion effect of miR-497 ectopic expression (Olympus DP70, magnification ×200). *P < 0.05.

Next, the mechanism by which miR-497 inhibited tumor metastasis was elucidated. Among the predicted targets of miR-497, we focused on AEG-1 (Figure 6A) because of its carcinogenicity in several cancers. MiR-497 down-regulation was correlated with the overexpression of AEG-1 in human HCC specimens (Figure 1C and 1D). Luciferase assay revealed that miR-497 directly suppressed the activity of luciferase reporter with wild-type 3′UTR of AEG-1 (Figure 6B). Moreover, the expression levels of AEG-1 mRNA and protein were both significantly decreased in Huh7 cells transfected with miR-497 compared with controls (Figure 6C and 6D). Consistently, the overexpression of AEG-1 in miR-497 transfectants obviously abrogated the inhibitory effects of miR-497 on cell migration and invasion (Figure 7). Together, the above results demonstrated that miR-497 suppresses HCC metastasis by directly targeting AEG-1.

**Figure 8:**
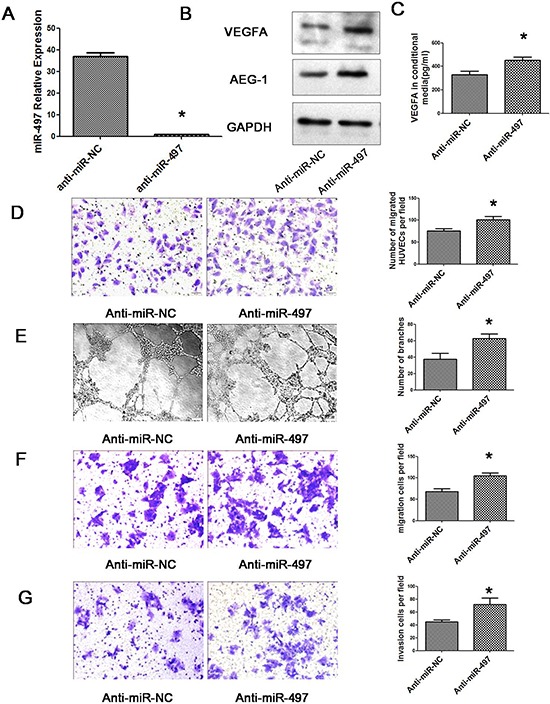
Down-regulation of miR-497 promotes angiogenesis, migration and invasion of HepG2 cells **A.** Relative expression of miR-497 detected by qRT–PCR in HepG2 cell lines transfected with anti-miR-497 or anti-miR-NC. The average miRNA expression in anti-miR-497 group was designated as 1. **B.** Down-regulation of miR-497 increased the protein levels of cellular VEGFA and AEG-1. HepG2 cells that were transfected with anti-miR-NC (lane 1) or anti-miR-497 (lane 2) for 48 h were analyzed by western blot assay. GAPDH was used as an internal control. **C.** The amount of secreted VEGFA was increased by inhibition of miR-497. Conditional medium from HepG2 cells that were transfected with anti-miR-NC or anti-miR-497 were analyzed by ELISA. **D.** Human umbilical vein endothelial cell (HUVEC) migration was evaluated using a 24-transwell chamber with 8 μm pore insert. The migration ability of HUVEC cells were significantly promoted by miR-497 down-regulation in HepG2 cells (Olympus DP70, magnification ×200). **E.** Inhibition of miR-497 facilitated the HCC cell–promoted HUVEC tube formation (Olympus DP70, magnification × 100). **F.** Down-regulation of miR-497 promotes HepG2 cell migration. HepG2 cells that were transfected with anti-miR-NC or anti-miR-497 were added to transwell chambers and incubated for 72 h, followed by staining with crystal violet (Olympus DP70, magnification × 200). **G.** Inhibition of miR-497 promotes HepG2 cell invasion. HepG2 cells that were transfected with anti-miR-NC or anti-miR-497 were added to transwell chambers with Matrigel coatings and incubated for 72 h, followed by staining with crystal violet (Olympus DP70, magnification ×200). *P < 0.05.

To further confirm the anti-angiogenic and anti-metastatic function of miR-497 in HCC cells, loss-of-function studies using anti-miR-497 were performed in HepG2 cells which express high levels of miR-497. As shown in Figure 8, down-regulation of miR-497 significantly increased the protein levels of cellular VEGFA and AEG-1 as well as the secretory levels of VEGFA in CM. Moreover, anti-miR-497 could not only promote the migration and tube formation ability of HUVEC cells, but also stimulate the migration and invasion ability of HepG2 cells (Figure 8).

### MiR-497 inhibits the tumor angiogenesis and metastasis of hepatoma xenografts in nude mice

**Figure 9:**
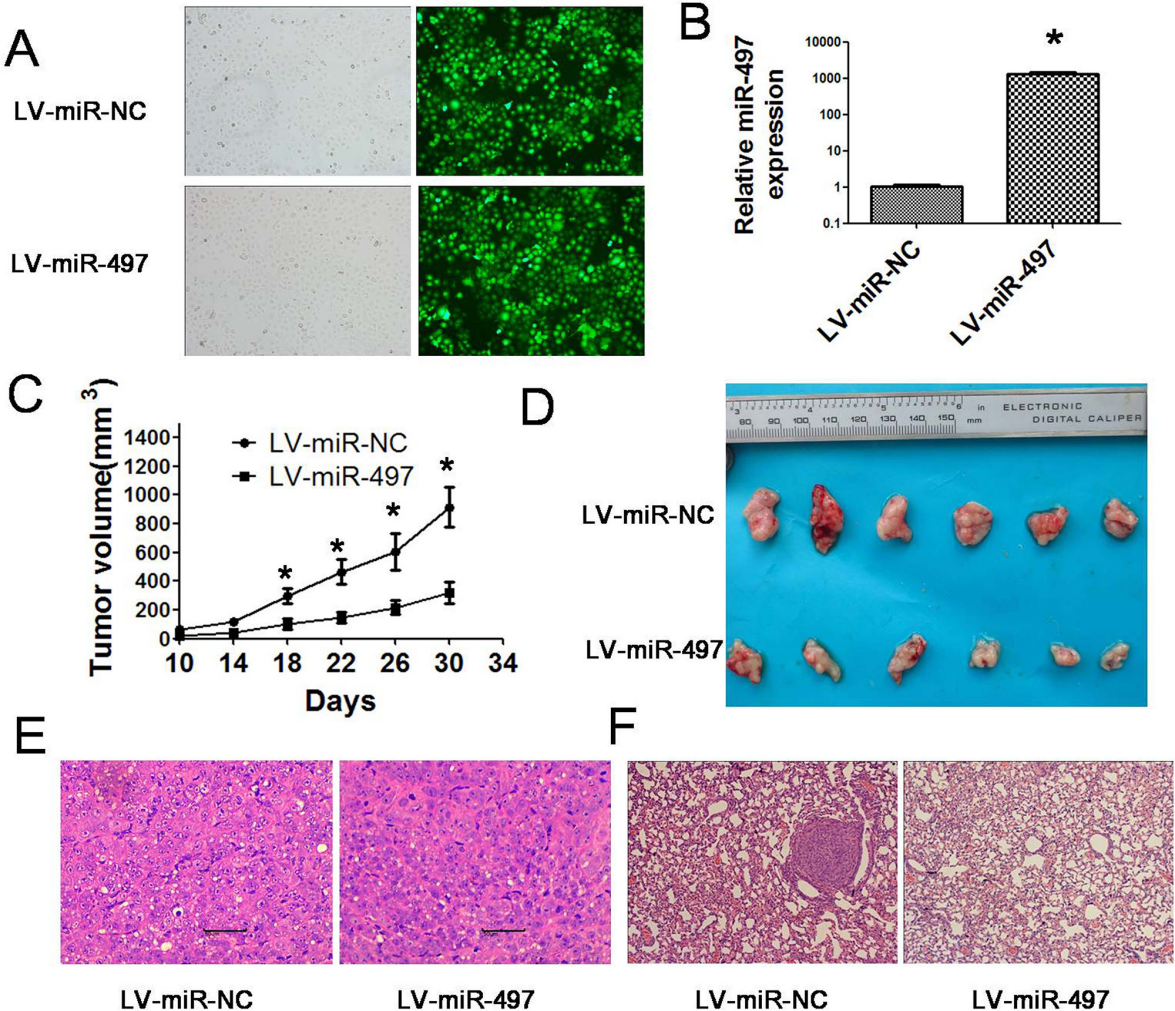
miR-497 impairs the growth of hepatoma xenografts and suppresses metastasis *in vivo* **A.** Efficient transfection of lentivirus in Huh7 cells was shown by using fluorescent microscopy. The black and white pictures showed the cells in the same field under normal white light. **B.** SYBR Green qRT–PCR was used to evaluate relative expression of miR-497 in Huh7 cells transfected with lentivirus LV-miR-497 or LV-miR-NC. The average miRNA expression in LV-miR-NC group was designated as 1. **C.** Huh7 cells transfected with lentivirus LV-miR-497 or LV-miR-NC were injected subcutaneously into nude mice. From the 10th day, tumor sizes were measured every four days and tumor growth curves were obtained. Data are presented as tumor volume in the mean ± SD. **D.** 30 days after the injection of Huh7 cells, mice were sacrificed and photographed. **E.** Hematoxylin-eosin staining was performed on serial sections of subcutaneous xenograft tumors. **F.** Hematoxylin-eosin staining was performed on serial sections of lungs to detect the metastatic nodules. Huh7 cells transfected with lentivirus miR-497 or miR-NC were injected into the tail vein of nude mice. Four weeks after injection, the mice were sacrificed and lungs were dissected, fixed in formalin, embedded in paraffin, and sectioned for hematoxylin-eosin staining. *P < 0.05.

**Figure 10:**
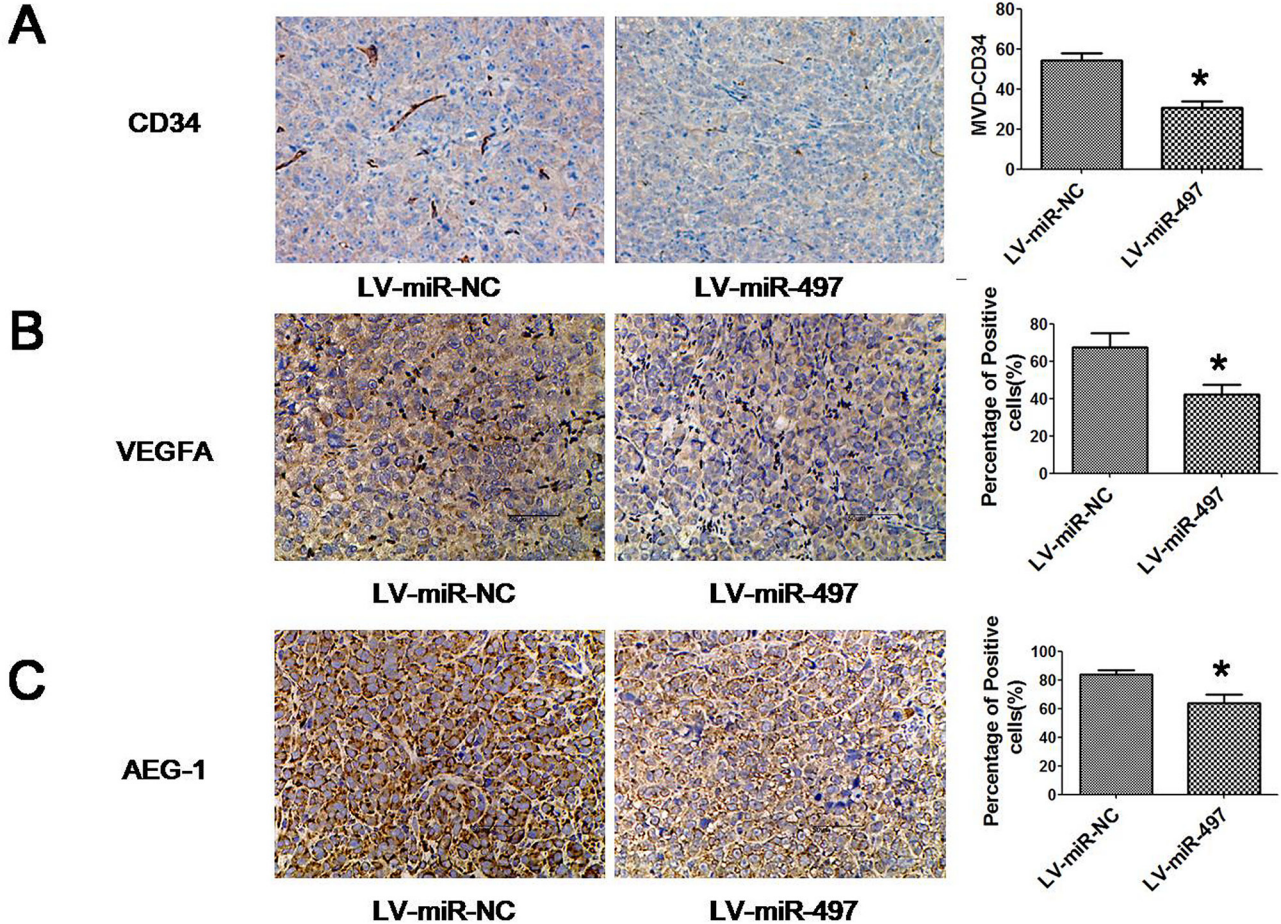
The expression of indicated proteins in xenograft tumors The expression of CD34 **A.**, VEGFA **B.** and AEG-1 **C.** was examined by immunohistochemical staining. Restoration of miR-497 expression reduced AEG-1, CD34 and VEGFA expression in xenograft tumors established by Huh7 cells. Representative immunohistochemical staining and the percentage of positive cells in immnostaining were shown. Positive cells were counted in tumor tissues and presented as the mean ± SD (4 random fields per section and three sections per tumor). *p < 0.05.

**Figure 11:**
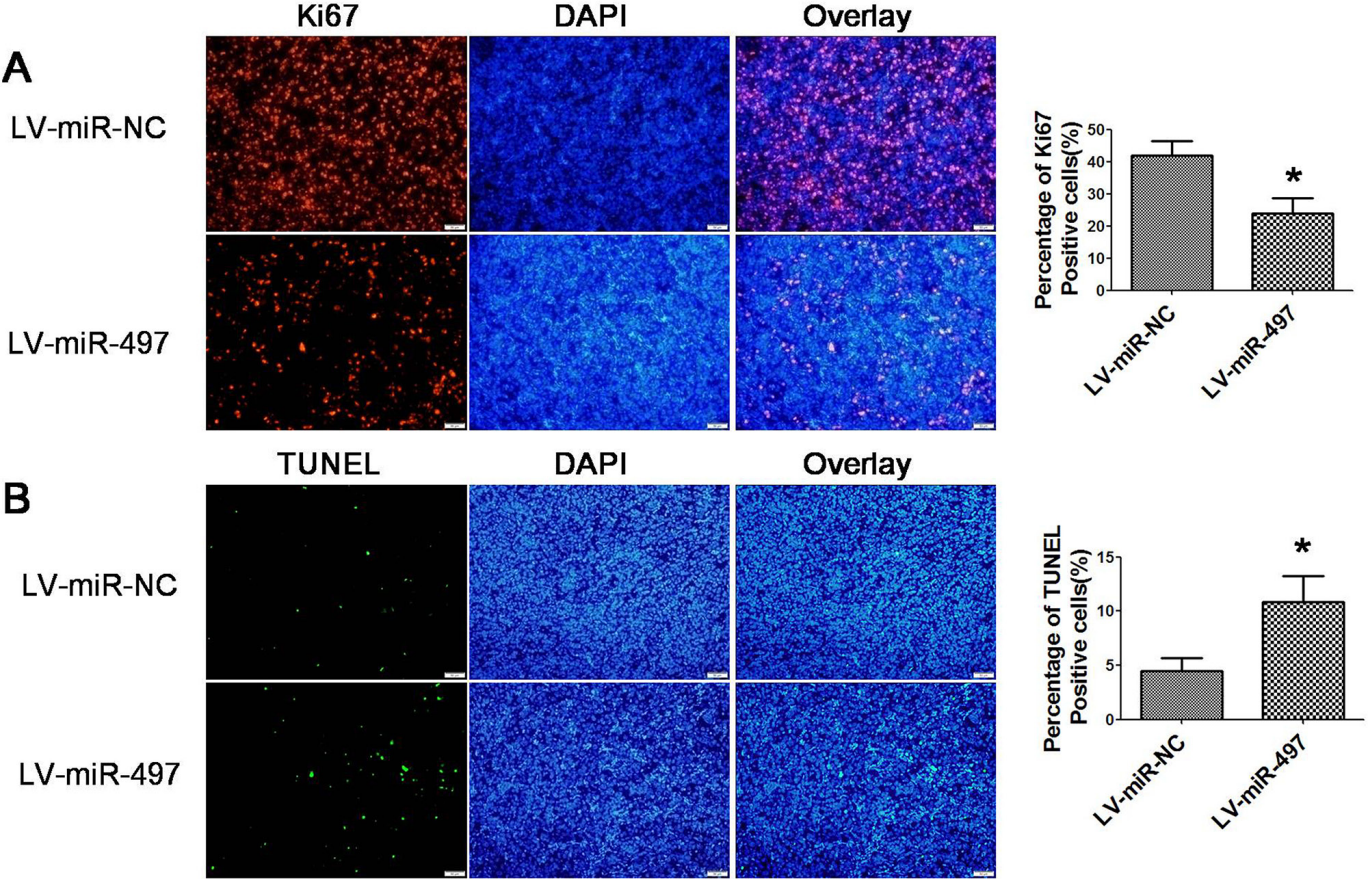
Effects of miR-497 on the proliferation and apoptosis *in vivo* **A.** Effect of miR-497 on the proliferation of xenograft tumors established by Huh7 cells. Cell proliferation was assessed by immnofluorescence staining of Ki67. Representative immunofluorescence staining and the percentage of ki67-positive cells were shown. **B.** Effect of miR-497 on the apoptosis of xenograft tumors established by Huh7 cells. The apoptosis status of tumor sections were evaluated using TUNEL assay. Representative pictures and the percentage of TUNEL positive cells were shown. *p < 0.05.

Considering the important roles of miR-497 in HCC, we next used HCC xenograft models to further confirm the above findings *in vivo*. Since miR-497 was significantly downregulated in HCC, we successfully constructed a recombinant lentiviral vector named LV-miR-497 (and LV-miR-NC as control) to transduce Huh7 cells (Figure 9A), and increase the expression of miR-497 (Figure 9B). Then, LV-miR-497 or LV-miR-NC transfected Huh7 cells were injected in the flanks of athymic nude mice to establish subcutaneous HCC xenograft. 30 days after injection, tumors were removed and measured. Tumor sizes in LV-miR-497 group were much smaller than that in LV-miR-NC group (Figure 9C, 9D and 9E; tumor incidence for LV-miR-497 versus LV-miR-NC groups: 6/6 versus 6/6). Additionally, LV-miR-NC or LV-miR-497 transfected Huh7 cells were injected into the tail vein of nude mice to assess the metastatic activity. Mice were sacrificed four weeks after inoculation, and the number of metastatic nodi in lungs was reduced in the nude mice injected with the LV-miR-497 transfected cells, when compared to the LV-miR-NC group (Figure 9F). To clarify the cellular mechanisms underlying miR-497 mediated tumor suppression, resected tissues from those subcutaneous xenograft tumors were analyzed to verify AEG-1, CD34 and VEGFA expression. As shown in Figure 10, the LV-miR-497 group displayed reduced AEG-1, CD34 and VEGFA expression in the tumor tissues. Furthermore, the expressions of Ki67 as markers of proliferation and TUNEL Staining assay for apoptosis were also detected in these resected tumor tissues. Consistently, LV-miR-497 group showed a status of proliferation inhibition and apoptosis enhancement compared with LV-miR-NC group (Figure 11). These data provide strong evidence that miR-497 could inhibit HCC angiogenesis and metastasis *in vivo*.

## DISCUSSION

Accumulating evidences indicated that dysregulation of miRNAs was frequently observed in multiple types of cancers and played fundamental roles in tumor initiation and progression [3, 17]. Reduced expression of miR-497 was found in multiple cancers including HCC [11]. Our data indicated that the expression of miR-497 in HCC tissues was significantly lower than that in matched adjacent non-tumor tissues, which is in agreement with a previous report [11]. Additionally, we found that the expression of miR-497 was also generally suppressed in 8 hepatoma cell lines compared with 2 normal hepatic cell lines. These results afford reference for the option of cell model for further research of miR-497 in HCC.

Angiogenesis is the process to generate tumor-associated neovasculature, which addresses the needs of tumor for nutrients and oxygen. In the healthy state, normal vasculature becomes largely quiescent [2]. In contrast, during tumor progression, an “angiogenic switch” is almost always activated and remains on, causing normally quiescent vasculature to continually sprout new vessels that help sustain expanding neoplastic growths [2]. VEGFA, which can be upregulated by oncogene signaling, was one of the well-known angiogenesis inducers [14, 15]. Our data indicated that HCC tissues displayed higher expression of CD34 (surface marker of neovascular endothelial cells) and VEGFA than their matched adjacent nontumor tissues. Loss-of-function analyses disclosed that inhibition of VEGFA could suppress HCC angiogenesis *in vitro*. These results were consistent with those in literatures [18, 19]. Previous studies have demonstrated that several miRNAs are involved in the regulation of HCC angiogenesis, for example, miR-195, miR-491, miR-126-3p, etc [20–22]. In this study, we have found the possibly effects of miR-497 in HCC angiogenesis for the first time. Both *in vitro* and *in vivo* data indicated that miR-497 was capable of suppressing HCC angiogenesis. Furthermore, we also demonstrated that miR-497 suppressed VEGFA expression by binding directly to the 3′-UTR of VEGFA. Wang W et al. have demonstrated that miR-497 suppresses angiogenesis by targeting VEGFA in ovarian cancer, which showed similar results to our findings [9]. These data indicated that miR-497 suppressed VEGFA expression and the negative regulation of VEGFA by miR-497 might contribute partially to anti-angiogenesis effects of miR-497 involved in HCC.

Frequent metastasis is another hallmark for malignant tumors, which is a difficulty for HCC therapy. The capability for invasion and metastasis enables cancer cells to escape the primary tumor mass and colonize new terrain in the body where, at least initially, nutrients and space are not limiting [23]. Previous researches have shown that miRNAs are implicated in the process of tumor invasion and metastasis. For instance, miR-29a/b was reported to enhance cell migration and invasion in nasopharyngeal carcinoma progression by regulating SPARC and COL3A1 gene expression [24]. MiR-375 was also confirmed to inhibit metastasis and invasion of HCC cells [13]. For miR-497, it was reported to inhibit ovarian cancer cell migration and invasion through targeting of SMAD specific E3 ubiquitin protein ligase and modulate gastric cancer cell invasion by repressing eIF4E [25, 26]. But the role of miR-497 in HCC metastasis has not been explored. Here, we disclosed that miR-497 could suppress HCC metastasis and invasion *in vitro*. Furthermore, *in vivo* analysis indicated that the induction of miR-497 expression markedly decreased the pulmonary metastasis of circulating HCC cells. AEG-1 was reported to be overexpressed in multiple types of cancers and promote cell metastasis and invasion [16, 27–29]. It has now become a recognized cancer promoter because that AEG-1 functions as a downstream mediator of the oncogenic c-Myc and Ha-Ras, and its overexpression activates the PI3K/Akt and Wnt/b-catenin signaling pathways [16]. In our study, inverse correlation was observed between miR-497 and AEG-1 expression in human HCC tissues, which had not been shown before. We also demonstrated for the first time that miR-497 suppressed AEG-1 expression by directly binding to the 3′-UTR of AEG-1. Moreover, restoration of AEG-1 could partially reverse the anti-invasion and anti-metastasis effects induced by miR-497. These results suggested that miR-497 suppressed AEG-1 expression, and the negative regulation of AEG-1 by miR-497 might contribute partially to anti-metastasis effects of miR-497 involved in HCC.

In conclusion, our findings indicate that miR-497 suppresses angiogenesis and metastasis of HCC cells *in vitro* and *in vivo* by inhibiting the expression of VEGF and AEG-1, and highlight the therapeutic potential of miR-497 in HCC.

## MATERIALS AND METHODS

### Cell lines and patient samples

The hepatoma cell lines HepG2, Huh7, PLC/PRF/5, SMMC-7721, SK-HEP-1, Hep3B, the human normal hepatic cell line L02 and human umbilical vein endothelial cell lineHUVEC were obtained from the China Center for Type Culture Collection (CCTCC). The human normal hepatic cell line chang liver was obtained from China Cell Culture Center (Shanghai). The HCC cell lines, MHCC-97H and MHCC-97L, used in this study were obtained from the Liver Cancer Institute, Fudan University (Shanghai, China). All the cells were cultured and maintained in DMEM medium (Gibco, USA) supplemented with 10% fetal bovine serum (Gibco, Grand Island, NY) and antibiotics (100 units/ml penicillin and 100 μg/ml streptomycin) in an atmosphere of 5% CO2 at 37°C. Following ethical and institutional guidelines and after informed consent of the tissue donors, samples (including 36 pairs of HCC and adjacent nontumor tissues) were collected from 36 HCC patients undergoing partial hepatectomy and immediately stored in RNAlater (Ambion, Austin, TX, USA). The characteristics of these patients are described in Supplementary Table S1.

### miRNA mimics, miRNA inhibitors, plasmid, siRNA and cell transfection

miR-497 mimics or inhibitors (anti-miR-497) and their matched negative controls (miR-NC or anti-miR-NC) were purchased from Guangzhou Ribobio Co., Ltd, China. VEGFA or AEG-1 overexpressing vectors (lacking 3′-UTR), VEGFA siRNAs (si-VEGFA) and their controls were prepared as previously described [13, 20]. Cell transfections were performed using Lipofectamine 2000 (Invitrogen, Carlsbad, CA, USA) according to the manufacturer's instruction. 50nM miRNA mimics or siRNA were used for transfection if not specifically mentioned.

### Collection of conditional medium (CM)

HCC cells tranfected with either miRNA mimics or siRNA or their matched control for 24 h were seed on six well plates at 2 × 10^6^/ml in DMEM supplemented with serum-free medium. After culture for 24 h, the supernatant was collected and centrifuged at 500 × g and 12,000 × g (4°C, 10 min each) to remove cell debris. Then aliquots of the CM were stored at −80°C until used.

### HUVEC recruitment assay

24-well Boyden chambers with 8-μm pore size polycarbonate membranes (Corning, USA) were used for endothelial recruitment assays. HCC cells were placed in the lower compartments and transfected with miR-497 mimics, anti-miR-497 or si-VEGFA or their matched negtive control for 36 h and refreshed with 600 μl serum-free medium before the recruitment experiments. Then HUVECs were resuspended in 100 μl of serum-free medium and seeded in the upper compartments of the chambers. After incubation at 37°C for 12 h, the cells remaining on the upper surfaces of the membrane were removed. The cells on the lower surfaces of the membrane were fixed, stained with crystal violet and counted under a light microscope.

### HUVEC capillary tube formation assay

HUVECs (3×10^4^) were grown in the absence or presence of 100% CM for 6 h at 37°C in a 96-well plate coated with Matrigel (BD Biosciences, USA). The formation of capillary-like structures was captured under a light microscope. The branch points of the formed tubes, which represent the degree of angiogenesis *in vitro*, were scanned and quantitated under a light microscope.

### *In vitro* tumor cell migration, invasion and growth assays

For tumor cell migration and invasion assays, 24-well Boyden chambers with 8-μm pore size polycarbonate membranes (Corning) were used. Huh7, PLC/PRF/5 or HepG2 cells were reverse transfected with miRNA mimics/inhibitors or (and) plasmid. For both the migration and invasion assays, cells were resuspended in 100 μl serum-free DMEM at 36 h post-transfection and were added to the upper compartments of the chambers, and the lower compartments were filled with 600 μl of DMEM with 15% FBS. For invasion assays, the membranes were coated with 60 μg of Matrigel to form matrix barriers in advance. After incubation at 37°C for 24 h (Huh7), 36 h (PLC/PRF/5) or 48 h (HepG2), remove the remaining cells on the upper surfaces of the membrane. Then cells on the lower surfaces of the membrane were fixed, stained with crystal violet and counted under a light microscope.

### Enzyme-linked immunosorbent assay

VEGFA in culture supernatants was detected by enzyme-linked immunosorbent assay (Dakewe Biotech Company, China) according to the manufacturer's instructions.

### SYBR Green qRT-PCR

RNA extraction was performed as described previously [13]. First-strand of complementary DNA was synthesized from 1 μg of total RNA using the First Strand cDNA Synthesis Kit (Fermentas, Burlington, VT, USA). Real-time PCR was performed by using the Platinum SYBR Green qPCR SuperMix UDG reagent (Invitrogen) according to the manufacturer's instruction. β-actin RNA was used as an internal control. The primer sequences of genes are listed in Supplementary Table S2. The expression levels of miR-497 were analyzed by Bulge-LoopTM miRNA qRT-PCR Primer (RiboBio, China) and normalized to U6. Data analysis was performed using the 2ΔΔCt method. Each sample was tested in triplicate.

### Western blot analysis

For each sample, total protein extracts were separated on SDS-PAGE gels and transferred to polyvinylidene fluoride membranes, which were blocked with 5% non-fat dry milk for 1 h and incubated with primary antibodies overnight at 4°C and then with secondary antibodies for 1 h at room temperature. The antibody for VEGFA (19003-1-AP) and AEG-1 (13860-1-AP) were obtained from Proteintech. Anti-GAPDH (sc-47778) and goat anti-rabbit IgG-horseradish peroxidase (sc-2004) were obtained from Santa Cruz. Band signals were visualized using an enhanced chemiluminescence kit (Pierce, Minneapolis, MN, USA).

### Luciferase assay

Double-stranded oligonucleotides corresponding to the wild-type (wt) or mutant (mut) miR-497 binding site in the 3′-UTR of VEGFA and AEG-1 were synthesized and subcloned into the pMIR-REPORT system (Applied Biosystems). Synthesized sequences are listed in Supplementary Table S3. In 96-well plates, Hela cells were cotransfected with 0.1 mg of pLUC-wt-gene or pLUC-mut-gene, 0.01 mg of pMIR-REPORT β-galactosidase plasmid served as an internal transfection efficiency control, and miR-NC or miR-497 mimic with a 50 nM final concentration. At 48 h after transfection, luciferase and β-galactosidase activities were measured using the Dual-Light System (Applied Biosystems).

### Immunohistochemistry

Serial sections (4 μm) were cut and then placed on glass slides. Deparaffinize slides in 2 changes of xylene, each for 10 minutes. Then rehydrate sections by sequentially incubating with 100%, 95%, 80% and 60% ethanol for 5 minutes each. For antigen retrieval, the sections were boiled in citrate buffer (pH 6.0) for 15 minutes in a microwave oven. Endogeneous peroxidase activity was blocked by incubation with 3% hydrogen peroxide solution for 10 minutes. Subsequently, the primary antibodies, rabbit-anti-human-VEGFA-antibodies (Proteintech, China), rabbit-anti-human-CD34-antibodies (Santa Cruze, USA) rabbit-anti-human-AEG-1-antibodies (Proteintech, China) were applied to the tissue sections and allowed to incubate overnight at 4°C. Then samples were further processed as per manufacturer's instructions and a series of procedures, including incubating with peroxidase-conjugated secondary antibody, staining, mild re-dyeing with hematoxylin, dehydration, coverslipping, and microscope observation, were performed in sequence.

### Lentivirus-based miR-497 overexpression

In order to elucidate the role of miR-497 *in vivo*, we constructed a recombinant lentivirus termed LV-miR-497 to generate stable gain-of-function of miR-497 in hepatoma cells. The recombinant lentivirus LV-miR-497 and its control LV-miR-NC were prepared as previously described [30].

### Nude mouse xenograft studies

BALB/c athymic nude mice (male, 5–6-weeks old and 16–20 g) were purchased from Beijing HFK Bioscience Co. LTD and were bred at pathogen-free conditions. All animal experiments were approved by Tongji Medical College Institutional Animal Care and Use Committee. The LV-miR-497 or LV-miR-NC transfected Huh7 cells were harvested from tissue culture flasks using trypsin and washed three times with PBS. Then cells (6 × 10^7^ cells per ml) were suspended in serum free DMEM. Mice were randomly divided into two groups, each containing 6 mice, and cells were subcutaneously injected (100 μl per mouse) the flank of each mouse (day 0). After 10 days, the length and width of tumors were measured every 4 days. Tumor volume (V) was monitored by measuring the length (L) and width (W) with vernier caliper and calculated with the formula V = (L × W^2^) × 0.5. On the 30th day after injection, mice were sacrificed and tumors were harvested and photographed.

### *In vivo* metastasis assay

For the *in vivo* metastasis assays, the LV-miR-497 or LV-miR-NC transfected Huh7cells were harvested from tissue culture flasks using trypsin and washed three times with PBS. Then 5 × 10^5^ cells were suspended in 100 μl PBS for each mouse (six in each group, male BALB/c athymic nude mice, 5–6-weeks old and 16–20 g) and injected into the tail vein. After four weeks of injection, the mice were sacrificed and lungs were dissected, fixed in formalin, embedded in paraffin, and sectioned for hematoxylin-eosin staining.

### Immunofluorescence staining and TUNEL staining

Tumor specimens were fixed in 4% paraformaldehyde, embedded in paraffin, cut into 4 μm pieces and mounted on polylysine-coated slides. Immunofluorescence staining and TUNEL staining were performed as we have described before [31, 32].

### Statistical analysis

All data are expressed as mean ± s.e. from at least three separate experiments performed in triplicate except otherwise noted. The differences between groups were analyzed by Student's *t* test and *P* < 0.05 was considered to be statistically significant.

## SUPPLEMENTARY FIGURES AND TABLES


