# Supplementary material for: MiR-497 suppresses angiogenesis and metastasis of hepatocellular carcinoma by inhibiting VEGFA and AEG-1
Source: Oncotarget. 2015 Aug 21;6(30):29527–42. doi: 10.18632/oncotarget.5012 (PMC4745744; doi:10.18632/oncotarget.5012)
Supplement: Supplementary file 1 [file oncotarget-06-29527-s001.pdf]

SUPPLEMENTARY FIGURES AND TABLES

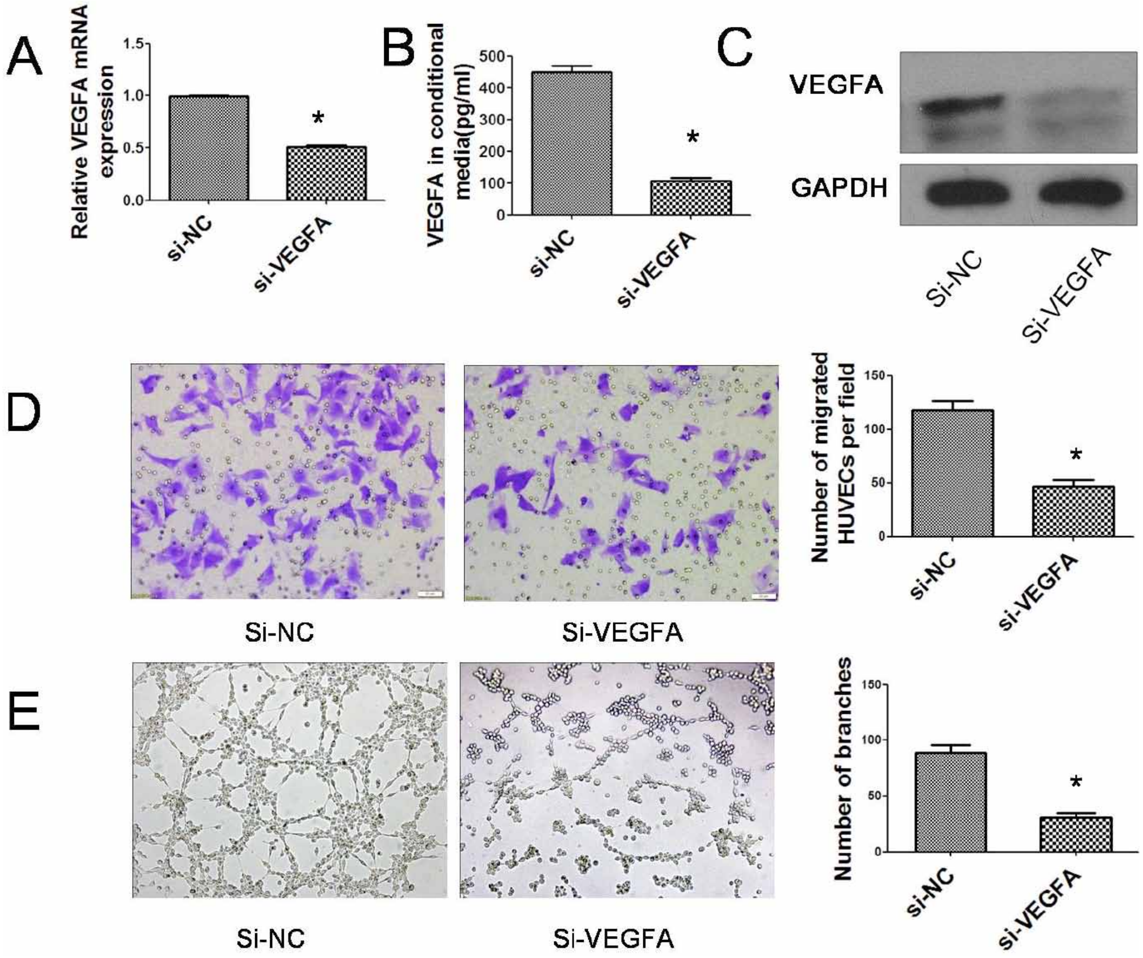

Supplementary Figure S1: VEGFA knockdown phenocopies miR-497-mediated anti-angiogenic ability in Huh7 cells. A. Relative mRNA expression of VEGFA were detected by qRT–PCR in Huh7 cells transfected with si-VEGFA or negtive control si-NC. The average mRNA expression in si-NC group was designated as 1. **B.** ELISA showed that the amount of secreted VEGFA was decreased by transfection of si-VEGFA in Huh7 cells. **C.** Western blot assay shows that transfection of si-VEGFA reduced the protein levels of endogenous VEGFA in Huh7 cells. **D.** The migration ability of human umbilical vein endothelial cells (HUVEC) was significantly inhibited by si-VEGFA transfection in Huh7 cells. (Olympus DP70, magnification ×200). **E.** Knockdown of VEGFA suppressed the HCC cell–promoted HUVEC tube formation (Olympus DP70, magnification ×100). $^{*}P < 0.05$.

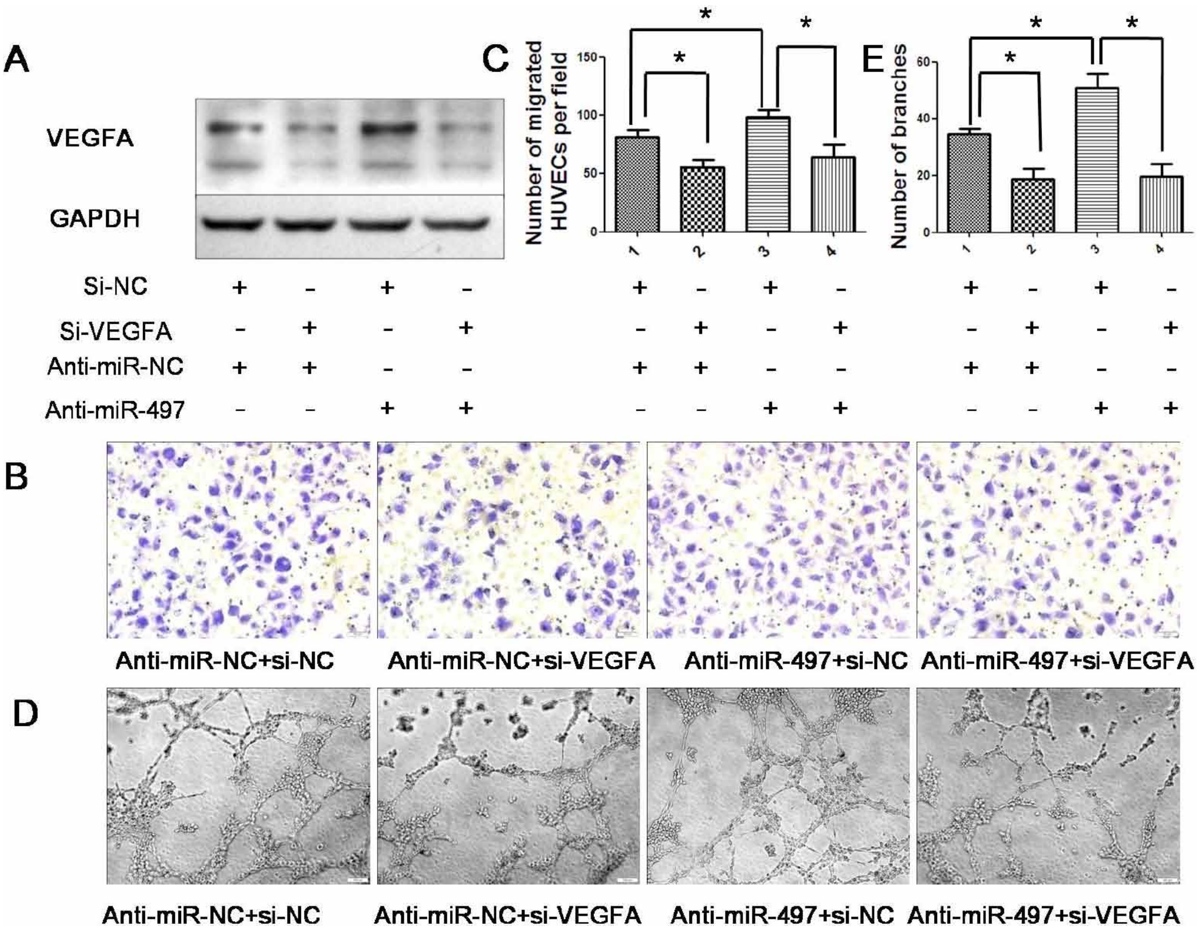

Supplementary Figure S2: Down-regulation of VEGFA attenuates the pro-angiogenic effect of anti-miR-497 in HepG2 cells. A. Forty-eight hours after co-transfected with anti-miR-NC or anti-miR-497 and si-NC or si-VEGFA, HepG2 cells were analyzed by immunoblotting. GAPDH was used as an internal control. **B.** Human umbilical vein endothelial cell (HUVEC) migration was evaluated using a 24-transwell chamber with 8 μm pore insert. The promotion of migration ability of HUVEC cells by miR-497 down-regulation was antagonized by VEGFA knockdown (Olympus DP70, magnification ×200). **C.** Quantification of the migration capability of HUVEC cells. **D.** Inhibition of VEGFA antagonized the pro- tube formation effect of anti-miR-497 (Olympus DP70, magnification ×100). **E.** Quantification of the tube formation capability of HUVEC cells. The results were reproduced in three independent experiments, and representative images are shown. *$P < 0.05$.

Supplementary Table S1: The characteristics of hepatocellular carcinoma patients included in this study

Clinicopathological parameters	Number (Frequency)
**Gender**	
**Male**	33 (91.7%)
**Female**	3 (8.3%)
**Age**	
**≥40 year**	33 (91.7%)
**<40 year**	3 (8.3%)
**HBsAg**	
**Positive**	31 (86.1%)
**Negative**	5 (13.9%)
**Serum AFP level (ng/ml)**	
**<400**	20 (55.6%)
**≥400**	16 (44.4%)
**Serum ALT level (U/L)**	
**<50**	21 (58.3%)
**≥50**	15 (41.7%)
**New AJCC staging**	
**Early stage (I, II)**	31 (86.1%)
**Late stage (III, IV)**	5 (13.9%)
**Size of tumor (length, cm)**	
**<5 cm**	3 (8.3%)
**≥5 cm**	33 (91.7%)

HBsAg, hepatitis B virus surface antigen; AJCC, The American Joint Committee on Cancer; AFP, alpha fetoprotein; ALT, alanine aminotransferase

Supplementary Table S2: Primers used in SYBR Green qRT-PCR

Gene	Primers	Sequence
β-actin	Forward Primer	CATGTACGTTGCTATCCAGGC
	Reverse Primer	CTCCTTAATGTCACGCACGAT
VEGFA	Forward Primer	AGGGCAGAATCATCACGAAGT
	Reverse Primer	AGGGTCTCGATTGGATGGCA
AEG-1	Forward Primer	AAATGGGCGGACTGTTGAAGT
	Reverse Primer	CTGTTTTGCACTGCTTTAGCAT

Supplementary Table S3: The sequences of 55-mer double-stranded oligonucleotides containing the predicted miRNA binding sites

Targeted Gene	Sequence
wt-VEGFA-FS	*CTAGT*GAATTGGATTCGCCATTTTATTTTTCTTGCTGCTAAATCACCGAGCCCG*A*
wt-VEGFA-RS	*AGCTT*CGGGCTCGGTGATTTAGCAGCAAGAAAAATAAAATGGCGAATCCAATTC*A*
mut-VEGFA-FS	*CTAGT*GAATTGGATTCGCCATTTTATTTTTCTTGTATCGAAATCACCGAGCCCG*A*
mut-VEGFA-RS	*AGCTT*CGGGCTCGGTGATTTCGATACAAGAAAAATAAAATGGCGAATCCAATTC*A*
wt-AEG-1-FS	*CTAGT*AGAAATTTGGAAGGCTATTCAGTGCTGCTTAGTGTAGCAGCTAATAATG*A*
wt-AEG-1-RS	*AGCTT*CATTATTAGCTGCTACACTAAGCAGCACTGAATAGCCTTCCAAATTTCT*A*
mut-AEG-1-FS	*CTAGT*AGAAATTTGGAAGGCTATTCATGTATCGATAGTGTAGCAGCTAATAATG*A*
mut-AEG-1-RS	*AGCTT*CATTATTAGCTGCTACACTATCGATACATGAATAGCCTTCCAAATTTCT*A*

The sequences highlighted by red color refer to predicted pairing nucleotide with the seeding sequences of corresponding miR-497. Italic letters refer to overhangs of restriction enzyme sites. wt, wide type; mut, mutant type; FS, forward sequence; RS, reverse sequence.
